# Spatio-Temporal Epidemiology of Viral Hepatitis in China (2003–2015): Implications for Prevention and Control Policies

**DOI:** 10.3390/ijerph15040661

**Published:** 2018-04-02

**Authors:** Bin Zhu, Jinlin Liu, Yang Fu, Bo Zhang, Ying Mao

**Affiliations:** 1 School of Public Policy and Administration, Xi’an Jiaotong University, 28 Xianning West Road, Xi’an 710049, China; binzhu2-c@my.cityu.edu.hk (B.Z.); liujinlin_xjtu@stu.xjtu.edu.cn (J.L.); 2 Department of Public Policy, City University of Hong Kong, Tat Chee Avenue, Kowloon, Hong Kong, China; 3 College of Management, Shenzhen University, Nanhai Ave 3688, Shenzhen 518060, China; yangfu6-c@my.cityu.edu.hk; 4 School of Human Settlements and Civil Engineering, Xi’an Jiaotong University, 28 Xianning West Road, Xi’an 710049, China; zhangbo0505@stu.xjtu.edu.cn

**Keywords:** viral hepatitis, spatio-temporal epidemiology, spatial autocorrelation, space-time scan, Moran’s I, HAV, HBV, HCV, HEV, China

## Abstract

Viral hepatitis, as one of the most serious notifiable infectious diseases in China, takes heavy tolls from the infected and causes a severe economic burden to society, yet few studies have systematically explored the spatio-temporal epidemiology of viral hepatitis in China. This study aims to explore, visualize and compare the epidemiologic trends and spatial changing patterns of different types of viral hepatitis (A, B, C, E and unspecified, based on the classification of CDC) at the provincial level in China. The growth rates of incidence are used and converted to box plots to visualize the epidemiologic trends, with the linear trend being tested by chi-square linear by linear association test. Two complementary spatial cluster methods are used to explore the overall agglomeration level and identify spatial clusters: spatial autocorrelation analysis (measured by global and local Moran’s I) and space-time scan analysis. Based on the spatial autocorrelation analysis, the hotspots of hepatitis A remain relatively stable and gradually shrunk, with Yunnan and Sichuan successively moving out the high-high (HH) cluster area. The HH clustering feature of hepatitis B in China gradually disappeared with time. However, the HH cluster area of hepatitis C has gradually moved towards the west, while for hepatitis E, the provincial units around the Yangtze River Delta region have been revealing HH cluster features since 2005. The space-time scan analysis also indicates the distinct spatial changing patterns of different types of viral hepatitis in China. It is easy to conclude that there is no one-size-fits-all plan for the prevention and control of viral hepatitis in all the provincial units. An effective response requires a package of coordinated actions, which should vary across localities regarding the spatial-temporal epidemic dynamics of each type of virus and the specific conditions of each provincial unit.

## 1. Introduction

Mankind has been fighting for hundreds of years against infectious diseases, which has resulted in a significant burden on communities across the global [1]. The United Nations set the 2030 agenda for Sustainable Development in 2015, defining an ambitious goal for infectious disease control and prevention. With a particular interest in a few kinds of diseases (Health Target 3.3), the UN proposed to fight against infectious diseases with specific actions across the economic and social dimension [2]. Evidently, to accelerate the achievement of this magnificent health target, certain kinds of infectious diseases, which have brought great harm to societies and their economies, have been identified as the priority among priorities.

Viral hepatitis, which is commonly transmitted through the fecal-oral route (types A and E) or exposure to infectious blood or fluids (types B, C and D), is a liver inflammation resulting from a viral infection. Due to the high incidence and severe consequences, viral hepatitis has been an international public threat for almost all countries, especially in Asia and Africa [3]. As hepatitis B and C can always cause cirrhosis or liver cancer, viral hepatitis is also a major cause of death, with about 1.45 million people being killed by different kinds of viral hepatitis infection per year [1]. Besides, viral hepatitis is also a growing cause of mortality among people living with HIV, with a large amount of AIDS patients showing co-infections with hepatitis B and C virus [3].

As the most populous country in the world, China has been threatened by and fighting against viral hepatitis for a long time. The Chinese government began to report the cases of viral hepatitis in the 1960s. Over the subsequent 30 years, hepatitis A and B dominated the viral hepatitis epidemics in China. However, reported cases of viral hepatitis other than type A and B have increased swiftly in recent decades, and as a result, the government decided to report viral hepatitis as A, B, Non-A and B, unspecified (clinical viral hepatitis cases which cannot be clearly detected as infected by which kind of virus) in 1990 [4]. In the middle 1990s, the incidence of hepatitis B began to surpass hepatitis A and became the most widespread hepatitis infection in China, while more and more cases of Non-A and B hepatitis were reported [5]. In order to systematically monitor and control the epidemics of viral hepatitis, the Chinese Center for Disease Control and Prevention (China CDC) decided to classify the reported cases of viral hepatitis as hepatitis A, B, C, E, and unspecified viral hepatitis, while it decided not to report hepatitis D infections as they usually present as co-infections with hepatitis B. Nowadays viral hepatitis is listed as the class B notifiable infectious disease in China (Specified in the Law of the People’s Republic of China on the Prevention and Treatment of Infectious Diseases [6]), which is under strict control and surveillance by the infectious disease reporting system developed by the China CDC. According to the latest China Health and Family Planning Statistical Yearbook (CHFPSY), the number of reported cases of viral hepatitis in 2015 ranked first (1,218,946 cases) and accounted for 40.01% of the reported cases (3,046,447 cases) among all 42 notifiable infectious diseases in that year.

Numerous researchers have studied the epidemiology of viral hepatitis in China. On the one hand, some studies have focused on the temporal trends of different types of viral hepatitis. Ren et al. [7] compared the epidemiology of hepatitis A and E from 2004 to 2014 and concluded their converse temporal trends in China. Zhang and Wilson [5] reviewed the national trends of hepatitis A, B, C, E at the national level and unspecified hepatitis and compared them with other 7 types of notifiable infectious diseases in China. Sumi et al. [8] analyzed and predicted the temporal trends of hepatitis A, B, C, E in Wuhan, which is a medium-scale Chinese provincial capital city. On the other hand, some scholars focused on the hepatitis epidemiology at a time point or among a short time period, exploring the age-specific, gender-specific or space-specific epidemiology of viral hepatitis in China. Lu et al. [9] compared the prevalence of hepatitis A, B, C, E in different age and gender groups based on the cross-sectional data of six regions in China. Zhang and colleagues [10] investigated and conduct laboratory hepatitis infection tests (hepatitis B surface antigen and anti-HCV first, if positive, then further test HBV DNA and HCV RNA) for 227,808 study participants and thus concluded the prevalence of HBV and HCV with gender, age, ethnic group and education level. Jia et al. [11] mapped the prevalence of HEV antibodies in the Chinese population based on the third National Viral Hepatitis Prevalence Survey (NVHPS III). Wang et al. [12] did a spatial cluster analysis of hepatitis C virus infection in China and detected the hot spots and cold spots of HCV infections in China.

To conclude, most of the previous studies either paid their attention to the temporal trends of viral hepatitis or focused on the investigation-based epidemics of viral hepatitis, but few did the analysis from the spatio-temporal perspective and systematically compared the epidemics of all the types of viral hepatitis. It is well known that the epidemiology of all the infectious diseases differs across space and also changes with time, only a spatio-temporal analysis can provide a complete understanding of the situation of viral hepatitis prevention and control in China. Besides, different types of viral hepatitis still share similarities in prevention and control measures despite their different transmission routes and health outcomes. Therefore, the aim of this study is to study and visualize the spatio-temporal epidemiology of different types of viral hepatitis in China. To be more specific, we conduct this study from three dimensions. First, this study explores the temporal trends of viral hepatitis. Second, this study investigates the spatial clusters of viral hepatitis in China. Third, this study compares the spatio-temporal epidemiology of viral hepatitis in China. Based on the results, we could systematically discuss the situation and policy implications for the prevention and control of viral hepatitis in China.

## 2. Data and Methods

### 2.1. Study Setting and Data Resources

Viral hepatitis is a reportable disease under the strict surveillance of China CDC. Health professionals are required to record and report viral hepatitis infections through the national infectious disease system within one day. There are institutionalized and standardized procedures to reexamine and check the results of first diagnosis and the final results will be collected by China CDC afterwards [13]. As mentioned above, China CDC only summarizes the reported cases of hepatitis A, B, C, E and unspecified to avoid duplicate counting of hepatitis B and D infections.

We obtained the provincial year-end data of the incidence rates of viral hepatitis infections and population in all the available years (2003–2015) from China Health Statistical Yearbook (CHSY) 2004–2013, and CHFPSY 2014–2016. The data of viral hepatitis from these two yearbooks were coherent and both collected through the surveillance system of China CDC. The research reports to the panel data of viral hepatitis at the 31 provincial units in China, excluding Hong Kong, Macau and Taiwan. Table S1 in the supplementary file shows the incidence of hepatitis A, B, C, E and unspecified in each provincial unit from 2003 to 2015. Table S2 in the supplementary file shows the population in each provincial unit from 2003 to 2015.

### 2.2. Time-Series Analysis

The time-series analysis demonstrates the temporal trends of hepatitis incidence rate (the number of new cases per 100,000 populations in each year) across the nation and also in each provincial unit. On the one hand, the box plots, which displays the basic statistics (e.g., median, minimum, upper quartile (Q3) and lower quartile (Q1)) and identifies the outliers, are used to exhibit the overall temporal trends of incidence of viral hepatitis infections. On the other hand, the study time (2003–2015) is divided into two equal periods, 2003–2009, 2009–2015. The growth rates of the incidence in each provincial unit over the two sub-periods are displayed. Besides, the chi-square linear by linear association test is conducted for the incidence of each type of viral hepatitis to identify the units which displayed significant linear trend.

### 2.3. Spatial Clustering Analysis

This study employs two complementary methods to identify the spatial clusters of different types of viral hepatitis: spatial autocorrelation analysis and space-time scan analysis [14]. Spatial autocorrelation analysis is a research methodology that helps to delineate the spatial link of research targets in adjacent geographical units, which indicates the extent to which the values are unevenly distributed [15]. It can be used to identify various types of spatial clusters based on the spatial weight matrix but could only focus on one certain time point. In contrast, the space-time scan statistic complements the previous methods by effectively utilizing both the time and spatial information, but it could only identify a limited type of spatial clusters.

#### 2.3.1. Spatial Autocorrelation Analysis

In this study, the indicator of Moran’s I, which has been widely used in many spatial autocorrelation studies, is adpoted to examine the time-space distribution patterns of different types of viral hepatitis in China [16,17,18,19,20]. In particular, there are the global and local Moran’s I, which reflect the spatial autocorrelation of research targets on the global and local scales respectively [21]. In our study, the global Moran’s I reveals the overall spatial autocorrelation in the whole country while the local one focuses on each provincial unit and its surrounding regions. Table S3 in the supplementary file displays the formulas of and detailed explanations for Global and Local Moran’s I.

Global Moran’s I is a value between 1 and −1. In such a spectrum, 0 indicates that the research targets are randomly distributed in the overall geographical space and no concentration of values is detected. A value approaching 1 indicates the concentration of similar values, namely, low incidence of viral hepatitis with low ones or high with high ones [22]. A value approximating −1 indicates the other way around, the concertation of high with low values in the adjacent geographical units. In this study, the global Moran’s I of viral hepatitis in China from 2003–2015 are calculated.

**Figure 1:**
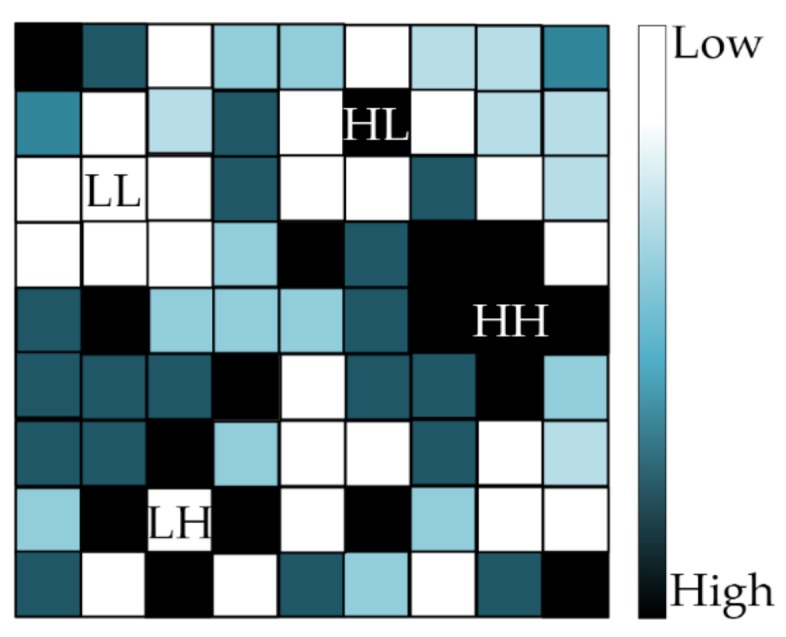
Four types of spatial clusters detected by local Moran’s I.

Local Moran’s I is used to indicate the spatial autocorrelation of each provincial unit with the surrounding units [22]. The value of local Moran’s I is also a number from −1 to 1 and the value can be explained exactly in the same way as that of the global Moran’s I. The function of local Moran’s I lies in the detection of spatial clusters (units whose local Moran’s I reached the significance level), which helps to reveal the spatial changing patterns of viral hepatitis across the research period. Figure 1 is an illustration of the spatial distribution of infectious diseases in the space in which each square represents a provincial unit and the color stands for the incidence. The local Moran’s I can detect four types of clusters, reflecting the high-low (HH, units with high incidence surrounded by units with high incidence, the same below), high-high (HL), low-low (LL) and low-high (LH) clustering patterns, respectively [23]. As this study covers a long time span, we selected three equidistant time points, namely 2003, 2009 and 2015, to reveal the spatial distribution pattern change throughout the time span.

For both global and local Moran’s I, Monte Carlo randomization (99,999 permutations) was employed to assess the significance of Moran’s I, with the null hypothesis being that the infected cases of viral hepatitis in China is completely random distributed [23].

#### 2.3.2. Space-Time Scan Statistics

The space-time scan statistic is defined by a cylindrical window with a circular geographical base which is centered on the centroids of areas, and with height corresponding to time. For this analysis, a discrete Poisson based model was used, where the number of cases in an area is Poisson distributed according to a known underlying population at risk [24]. The null hypothesis assumed that the relative risk (RR) of the incidence was the same within the window as compared with outside. The difference of the incidence inside and outside the windows was evaluated by the Log Likelihood Ratio (LLR):

$$\mathrm{LLR}=\log\left\{{\left(C/n\right)}^{c}{\left[\left(C-c\right)/\left(C-n\right)\right]}^{\left(C-c\right)}\right\}$$

where *C* denotes the total number of cases; *c* is the number of observed cases inside the window; *n* is the number of expected cases inside the window.

The space-time scan statistic is used to identify the most likely clusters (the window with largest LLR value) and secondary clusters (other windows with statistically significant LLR) and also the cluster time [25]. Statistical significance was evaluated in the Monte Carlo simulation method (replications set to 999 and significance level set at 0.05). Regarding the other parameters, the maximum radius of circular base was set at 50% of the total population at risk and the maximum height of the cylinder was set at 50% of the total study period.

### 2.4. Software Tools

The global Moran’s I and local Moran’s I were measured using the software GeoDa (Version 1.8.61, the University of Chicago, Chicago, IL, USA). The box plots were made with the Microsoft Excel (Version 2016, Microsoft Corp., Redmond, WA, USA). The chi-square linear by linear association test was conducted in SPSS (Version 20.0, IBM Inc., Armonk, NY, USA). The spatial-scan statistics were calculated with the SatScan (Version 9.5, Kulldorff and Information Management Services, Inc., Boston, MA, USA). All the maps were developed with software ArcGIS (Version 10.0, ESRI Inc., Redlands, CA, USA).

## 3. Results

### 3.1. Epidemiologic Trends

**Figure 2:**
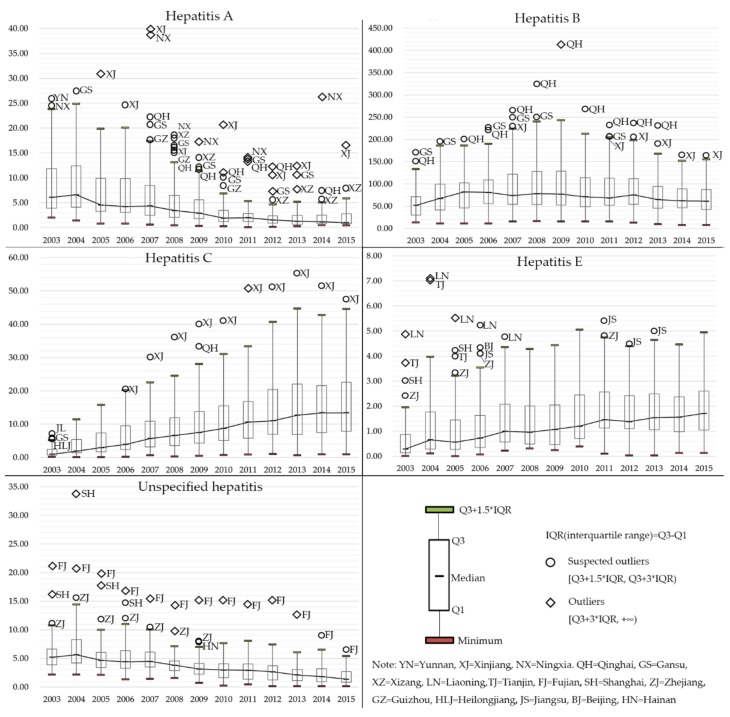
Box plots of the incidence of different types of viral hepatitis from 2003 to 2015.

Figure 2 shows the temporal trends of each type of viral hepatitis in box plots, with the black line connecting the median value in each year. The suspected outliers and outliers were identified and marked on the box plots. Evidently, different types of viral hepatitis displayed distinct epidemiologic trends. The upper quartile, median and lower quartile of the incidence of hepatitis A experienced a dramatic decrease since 2004, while the outliers mainly distributed in west China. As for hepatitis B, its median incidence remained relatively stable, while the IQR (interquartile range, the difference between the upper and lower quartile) gradually reduced after 2009. The temporal trends of hepatitis C were the other way round, the median, upper and lower quartile all increased dramatically across the research period, Xinjiang has been identified as a suspected outlier or an outlier since 2006. Similarly, the median, upper and lower quartile of the incidence of hepatitis E displayed a growing trend during 2003–2015. In contrast, it was easy to conclude that the basic statistics of the incidence of unspecified hepatitis displayed a downward trend, with Fujian showing a relatively high incidence of unspecified hepatitis across the whole research period.

To better understand the temporal trends of viral hepatitis in China, we also calculate the growth rate of the incidence of viral hepatitis in each provincial unit in Table 1, in which the incidence and growth rate across two sub-periods (2003–2009, 2009–2015) can be seen. Those provincial units which displayed a significant linear trend were in bold. Table S4 in the Supplementary File shows chi-square and *p*-value results for chi-square linear by linear association test. In general, the incidences of viral hepatitis in most provincial units were consistent with the general trend, while the growth rates of viral hepatitis in some provincial units differed greatly and even displayed a reverse trend. For example, the incidence of hepatitis A in Shanxi Province increased 43% during 2009 and 2015, which was in stark contrast to the −50% average growth rate across all the provincial units. Another example is the incidence of hepatitis C in Hainan Province increased 393% in the second sub-period, which was significantly higher than the average growth rate of 54%.

### 3.2. Global Spatial Autocorrelation

Table 2 shows the global spatial autocorrelation of all types of viral hepatitis and their test results. For Hepatitis A, B, C, E, their global Moran’s I all reached the significance level of 0.05 during the whole research period. In general, the global Moran’s I for hepatitis A was higher than 0.4 in most years, indicating a strong spatial cluster tendency of the reported cases. While the global Moran’s I for hepatitis B, C and unspecified hepatitis displayed a downward trend during 2003 to 2015. In contrast, the global Moran’s I for hepatitis E increased dramatically from 0.2093 in 2003 to 0.4378 in 2010, but the trend reversed and the global Moran’s I of hepatitis E ended up as 0.3572 in 2015.

### 3.3. Local Spatial Autocorrelation

**Figure 3:**
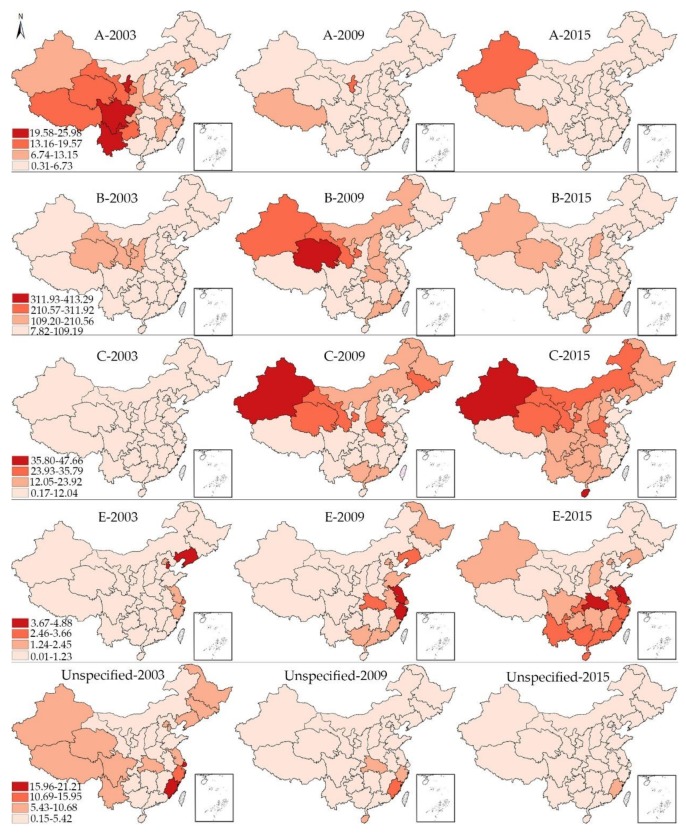
The hierarchy maps of the incidence rate of all types of viral hepatitis in 2003, 2009, 2015.

As the local spatial autocorrelation analysis only reveals the relative state rather than the absolute incidence in each provincial unit, we divide all the 31 provincial units in China into 4 classes in hierarchy maps (Figure 3) based on the maximum and minimum of one certain type of viral hepatitis during 2003, 2009 and 2015. The darker is the red color, the higher is the incidence of viral hepatitis. Take the hepatitis A as an example, the maximum and minimum of hepatitis A in 2003, 2009 and 2015 are 25.98 and 0.31, respectively. Then the difference between these two values are divided evenly into four categories.

**Figure 4:**
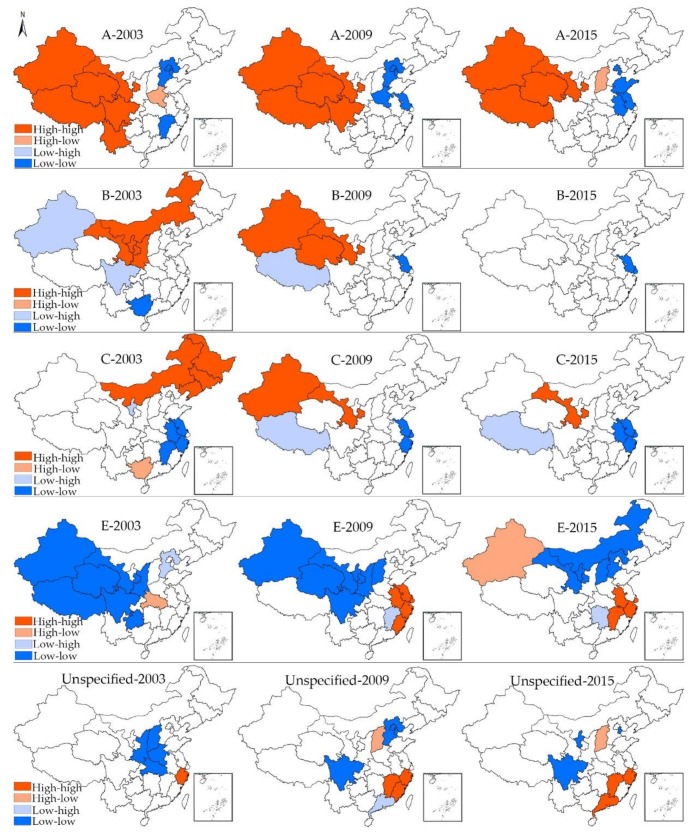
Spatial clusters of all type of viral hepatitis in 2003, 2009, 2015.

Figure 4 shows the spatial clusters for all types of viral hepatitis in 2003, 2009, and 2015, which reveals the spatial changing patterns of viral hepatitis in China. Only those units whose local Moran’s I have reached the significance level of 0.05 were displayed on the maps. For hepatitis A in 2003, all the HH clusters were located in west China, namely, Xinjiang, Xizang, Qinghai, Gansu, Sichuan and Yunnan. While Beijing, Tianjin, Hebei and Jiangxi displayed LL cluster feature and Henan displayed HL cluster feature. During the first sub-period (2003–2009), Yunnan moved out of the HH cluster area, while Henan and Jiangsu began to display LL cluster feature. When it comes to 2015, only four provincial units (Xinjiang, Xizang, Qinghai, Gansu) displayed HH feature. In addition, Shanxi was the only unit which displayed HL cluster feature. For hepatitis B, the HH cluster area gradually moved from north China (Neimenggu, Shaanxi, Ningxia and Gansu) to northwest China (Xinjiang, Qinghai, Gansu) during the first sub-period, while there were no provincial units displaying HH cluster feature in 2015, indicating a relatively average distribution of hepatitis B cases. In comparison, there is no law to follow for the changing pattern of LL and LH clusters, only Jiangsu displayed LL cluster feature at more than one time point.

As for hepatitis C, the HH cluster area was mainly concentrated in northeast China (Heilongjiang, Jilin, Liaoning, Neimenggu) in 2003. From 2003 to 2009, the HH cluster area moved towards the west, with Gansu and Xinjiang displaying HH cluster features in 2009, whereas only Gansu displayed HH cluster features in 2015. The LL cluster area mainly concentrated in the Yangtze River Delta region and remained relatively stable. Regarding hepatitis E, the most noticeable characteristic is that hotspots gradually emerged in Jiangsu, Anhui, Shanghai, Zhejiang and Fujian during the first sub-period, and these hotspots remained relatively stable after 2009, indicating the severe epidemics of hepatitis E in coastal areas. It is also important to note that Xinjiang turned from LL cluster type into HL cluster feature during 2009 to 2015, which indicates the disruptive changes of the epidemics of hepatitis E in Xinjiang. In addition, the LL cluster areas of hepatitis E also moved towards the east during the second period. At last, the HH cluster area of unspecified hepatitis mainly concentrated in southeast China (Zhejiang in 2003, Zhejiang, Fujian, Jiangxi in 2009, Zhejiang, Jiangxi, Guangdong in 2015), while Shanxi was the only unit which persistently displayed HL cluster feature during the second sub-period.

### 3.4. Space-Time Scan Analysis

**Figure 5:**
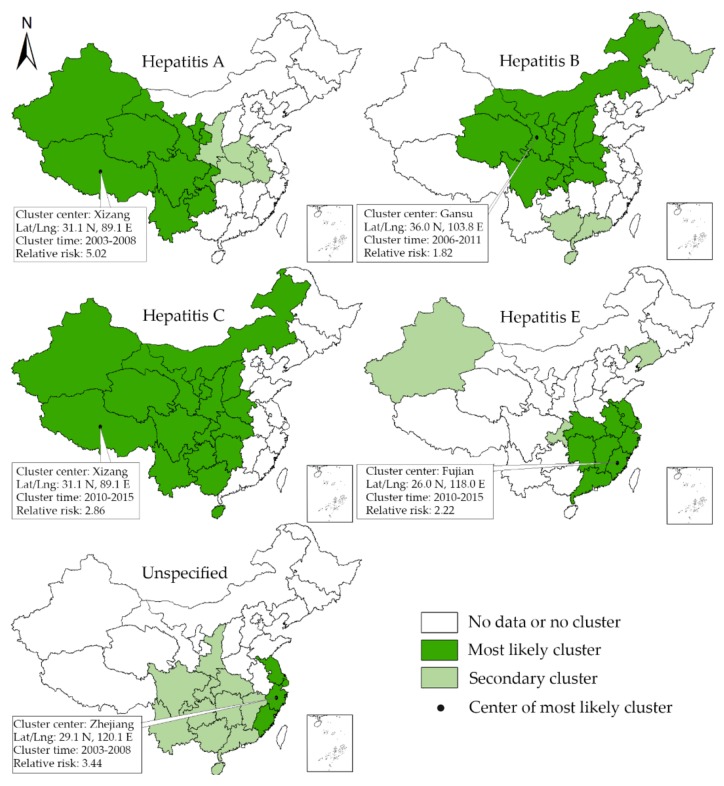
The spatial-temporal clusters detected by the space-scan statistics.

The results of space-scan analysis are shown in Figure 5. From 2003 to 2015, the space-scan statistic identified the most likely clusters for each type of viral hepatitis and 8 secondary clusters. The units which were included in most likely clusters of hepatitis A were concentrated in west China from 2003 to 2008, indicating the more severe epidemics of hepatitis A in this region. Regarding hepatitis B, many provincial units in central and west China were detected to be included in the mostly likely clusters from 2006 to 2011. In contrast, the most likely cluster area of hepatitis C was concentrated in middle and west China from 2010 to 2015. The most likely cluster areas of hepatitis E and unspecified hepatitis were concentrated in southeast coastal areas. More details about the most likely clusters and secondary clusters are shown in Table 3.

## 4. Discussion

This study conducted a comparative spatial-temporal epidemiology of different types of hepatitis viruses (A, B, C, E and unspecified), which provided much evidence for making area-targeted hepatitis prevention and control strategy in China. We would like to discuss the spatial-temporal epidemiology for different types of viral hepatitis first and then focus on the future prevention and control strategy.

Globally, China is still facing the severe threats of all types of viral hepatitis, with the reported cases of each kind of viral hepatitis distributed in all the provincial units. The global Moran’s I of the incidence of hepatitis A, B, C, E all reached the 0.05 significance level at all the time points, indicating the spatial concentration of the reported cases, which met our expectations and echoes the previous studies on infectious diseases distribution [12], while it is obvious that the spatio-temporal epidemiology of different types of hepatitis viruses (A, B, C, E and unspecified) in China are quite different, with different epidemiologic trends affecting different locations and revealing different spatial changing patterns.

On the one hand, the fecal-oral transmitted hepatitis forms (A and E) showed opposite trends and distinct spatial distribution characteristics. The incidence of hepatitis A was on a declining curve from 2003 to 2015, with the high-high cluster area remaining relatively stable and gradually shrinking. During 2003 to 2015, Yunnan and Sichuan successively moved out the high-high cluster area. In 2015, the high-high cluster area was mainly situated in the undeveloped areas, namely, Xinjiang, Xizang, Qinghai, Gansu. The space-time scan analysis also indicated the severe epidemics of hepatitis A in earlier years. This pattern can be attributed to two major factors. First, in the 1990s, there was a massive campaign to promote the vaccination of HAV. The HepA-L (Hepatitis A Attenuated Live Vaccine) and, HepA-I (Hepatitis A Inactivated Vaccine) are both listed in the class A vaccines of the national immunization program (the national immunization program includes two classes vaccines, for class A vaccines, all the citizens are duty-bound to vaccinate and can be vaccinated for free, while the class B vaccines are at citizens’ own expense, people can choose vaccine or not based on their own free will). Second, the overall sanitation and hygienic conditions in China have been improving substantially, breaking the contamination cycle of viral hepatitis, which also helps to explain why the high-high cluster area mainly concentrated in the undeveloped west China. Owing to the relatively underdeveloped economy and worse living conditions, west China is confronted with more difficulties to ensure the food and water safety. Therefore, it is still important to keep promoting the access to clean food and water as well as the hygienic living environment in undeveloped areas to contain the spreading and epidemics of type A hepatitis. In contrast, it is seemingly that the epidemics of type E hepatitis, whose incidence kept rising during the research period, has not been tamed as well. Therefore, it is believed that hepatitis E has not received due attention as other diseases posing a comparable burden of disease, such as HAV and HBV. Regarding its hotspots, HH or HL clusters are not widespread in 2003 yet later the Yangtze River Delta region began to display high-high cluster feature until now. It is also echoed by the spatial-scan analysis. In China’s Context, hepatitis E infection occurred, under most circumstances, as sporadic cases and occasional food-borne outbreaks, which results from poor sanitation conditions, including contamination of water and food from animal reservoir and human [11]. In consideration of the fact that the HH clusters of Hepatitis were mainly distributed in the Yangtze River Delta region, which is one of the three biggest integrated and dynamic city-regions in China, it is reasonable to recommend a stricter food and sanitation supervision in big cities. Beyond that, as effective vaccines also exist for hepatitis E infections [3], it is not unrealistic to introduce targeted vaccination campaign owing to the fairly strong economy and medical conditions in coastal areas.

On the other hand, the blood-borne transmitted hepatitis forms (B and C) also differ in epidemiologic trends and spatial changing patterns. Hepatitis B displayed a relatively stable status during the past decade. There were almost no significant clusters detected by spatial autocorrelation analysis and space-time scan analysis, indicating the effective control of the epidemics of hepatitis B in recent years. It is widely believed that the progress mainly results from the implementation of HBV vaccine programs in China [10,26,27]. Up to now, the childhood hepatitis B vaccination (three-dose, 0-1-6-month schedule) has been listed in class A vaccines of the national immunization program with the coverage rates of birth-dose and three-dose hepatitis B vaccine being both higher than 90% [28]. However, the universal newborn vaccination is not enough for the control of hepatitis B in China. Up to now, there is not yet a united strategy, either a plan or coverage by the health insurance [29], for hepatitis B vaccine among adults and adolescents older than 14 years old who have not been vaccinated. This results in low active hepatitis B vaccination rate among adults [29]. Besides, the measures to protect the most-at-risk populations (MARPs) should be strengthened. China still faces the challenge of mother-to-child transmission of hepatitis B [30,31]. In addition, in the contexts of growing mobility and increasingly open attitudes towards sex, the young people and adolescents, men who have sex with men (MSM), female sex workers (FSW), drug users and also the mobile populations should be paid more attention [32,33]. In particular, the epidemics of hepatitis D should also be taken seriously as about one-tenth of HBV infections are simultaneously infected with HDV in China [34]. In contrast to Hepatitis B, the epidemics of hepatitis C became much more severe from 2003 to 2015. In 2003, the prevalence level of hepatitis C was quite low, the high-high cluster area mainly concentrated in Northeast China, while with the increase of its incidence, the high-high cluster area began to move towards the west. In 2015, the median incidence among all the provincial units has exceeded 10/100,000 and only Gansu displays high-high cluster feature, indicating serious epidemics of hepatitis C in Gansu and its surrounding areas. While, it is also important to note that the epidemics of hepatitis C in Hainan should also be paid much attention as it had become the second highest in 2015 (Due to the boundary-based strategy of spatial weight matrix in this study, Hainan cannot be identified each type of cluster as it does not border with other units). In addition, the space-scan analysis also indicates the severe epidemics of hepatitis C in west China during 2010 to 2015. Now, HCV infection has not received enough attention in China, with a lot of people unaware of their infection, which potentially accelerated the transmission of hepatitis C [35]. Even the mandatory HCV screening has been implemented in blood donors control the HCV transmission in blood or blood product transfusion. However, it is believed that the paid blood donors, patients on hemodialysis, patients with hemophilia are still at higher risk of hepatitis C virus infection [36]. In particular, Since HBV and HCV can both be transmitted through the exchange of body fluids, HCV may either be co-infected with HBV and HIV, or both, thus sharing similarity in MARPs in China, such as the drug users and MSMs [37,38,39,40].

The unspecified viral hepatitis displayed a downward trend, which was mainly contributed to the development of diagnostic techniques [41]. Now, the diagnostic techniques for hepatitis B have been well-rounded, which makes it easy to diagnose. The diagnosis for hepatitis A and C is also easy with the combination of their clinical characteristics. Therefore it is believed that the unspecified viral hepatitis in China is mainly hepatitis E or other viruses, which should once again arouse our attention to tackle the epidemics of hepatitis C in China [4]. Anyhow, the accurate diagnosis of viral hepatitis is crucial for the treatment and epidemics control in latter stages, a powerful laboratory and diagnostic system vis-a-vis each type of viral hepatitis matters a lot in the reporting and treatment of infected cases.

Despite the widespread reported cases of all types of viral hepatitis, the number of deaths and morbidity due to viral hepatitis is decreasing with the development and treatment, which makes viral hepatitis fade away from the eyes of policy makers. Now it is quite clear about the measures to prevent and control each type of viral hepatitis. Recently in 2016, WHO released the strategies to deal with viral hepatitis infections worldwide, which partially contributes to the fulfillment of the 2030 SDGs [3]. As a national response, the central government of PRC has launched a program named National Prevention and Control Plan, led by the National Commission of Health and Planning and 10 other departments and ministries. While the prevention and control of viral hepatitis is a complex job owing to the various epidemiologic trends and spatial distribution characteristics. Therefore, it is suggested that every provincial unit should make its evidence-based plan which identifies the priority measures and enables meaningful inputs from all key stakeholders, such as the health sector, food sector, CDC, and so on. For those adjacent provincial units displaying HH or HL cluster features in 2015, it could more efficient if they can collaborate and formulate some joint measures. In particular, the measures to tackle viral hepatitis in China should not be limited to its prevention and control. Different from HAV and HEV, whose infections tend to be acute rather than chronic, HCV and HBV infections often cause chronic hepatitis and may develop into cirrhosis and hepatocellular carcinoma, which always require lifelong treatment. Patients infected with hepatitis B and C suffer from lifelong medical treatment, deteriorating their social and economic conditions even with the coverage of social and medical welfare [42]. Therefore, the government should not only ensure safe and effective prevention, care and treatment services but also their affordability together with multiple health security policies, such as health insurance and medical aids.

## 5. Conclusions

There is still a long way to go for the prevention and control of viral hepatitis in China. Owing to the distinct epidemiologic trends of viral hepatitis in each provincial unit, it is easy to conclude that there is no one-size-fits-all plan for the prevention and control of viral hepatitis in all the provincial units. An effective response requires a range of coordinated actions, which should vary across space based on the spatial-temporal epidemic dynamics of each type of virus and context in each provincial unit. In addition, to ensure a coherent rather than fragmented public health response it requires the government to coordinate among government factors, medical, food, insurance etc. and clarify stakeholder responsibility and accountability [3].

## Figures and Tables

**Table 1:** Growth of incidence of viral hepatitis in China and linear test (1/100,000) ^1^.

Region	Hepatitis A		Hepatitis B		Hepatitis C		Hepatitis E		Unspecificied Hepatitis	
	03^growth^09	09^growth^15	03^growth^09	09^growth^15	03^growth^09	09^growth^15	03^growth^09	09^growth^15	03^growth^09	09^growth^15
Beijing	3.69^(−75%)^**0.92	0.92^(−46%)^0.50	16.48^(37%)^22.54	22.54^(−65%)^**7.82	1.79^(393%)^*8.83	8.83^(−54%)^**4.07	1.56^(49%)^2.32	2.32^(−45%)^**1.28	7.59^(−86%)^**1.10	1.10^(−86%)^**0.15
Tianjin	2.11^(−85%)^**0.31	0.31^(39%)^0.43	23.05^(−11%)^20.59	20.59^(−39%)^**12.51	**1.42^(138%)^3.38**	3.38^(19%)^4.02	3.76^(−62%)^*1.42	1.42^(−54%)^**0.66	8.66^(−80%)^**1.75	1.75^(−71%)^**0.50
Hebei	3.12^(−64%)^**1.13	1.13^(−37%)^*0.71	57.57^(42%)^**82.00	82.00^(−10%)^73.94	0.73^(649%)^**5.47	5.47^(133%)^**12.74	0.56^(107%)^1.16	1.16^(−10%)^1.04	3.53^(−54%)^**1.62	1.62^(−43%)^**0.93
Shanxi	3.82^(−44%)^**2.14	2.14^(43%)^**3.05	30.95^(305%)^**125.34	125.34^(−1%)^124.54	1.62^(753%)^**13.82	13.82^(69%)^**23.31	0.20^(90%)^0.38	0.38^(334%)^**1.65	2.85^(32%)^3.76	3.76^(−39%)^*2.31
Neimenggu	5.90^(−66%)^**2.00	2.00^(−61%)^0.79	73.06^(81%)^**132.48	132.48^(−34%)^**87.94	3.33^(472%)^**19.04	19.04^(45%)^**27.52	0.23^(35%)^0.31	0.31^(142%)^**0.75	2.47^(−40%)^1.47	1.47^(−76%)^**0.36
Liaoning	11.46^(−71%)^**3.36	3.36^(32%)^4.44	36.72^(76%)^**64.52	64.52^(−30%)^**44.87	4.95^(161%)^**12.92	12.92^(55%)^**20.05	4.88^(−41%)^*2.88	2.88^(−23%)^*2.21	7.63^(−30%)^**5.36	5.36^(−32%)^**3.62
Jilin	6.33^(−74%)^**1.64	1.64^(−51%)^**0.81	46.03^(42%)^**65.18	65.18^(−37%)^40.86	7.65^(250%)^**26.74	26.74^(−13%)^23.17	0.79^(33%)^1.05	1.05^(0%)^1.05	6.95^(−39%)^**4.24	4.24^(−78%)^**0.94
Heilongjiang	4.74^(−78%)^**1.03	1.03^(−38%)^**0.64	57.27^(−8%)^52.92	52.92^(−50%)^**26.69	5.49^(152%)^**13.84	13.84^(−8%)^12.77	0.85^(64%)^1.39	1.39^(−21%)^**1.10	5.74^(−35%)^**3.74	3.74^(−48%)^**1.93
Shanghai	3.83^(−66%)^**1.29	1.29^(−37%)^0.81	16.87^(79%)^30.20	30.20^(15%)^34.74	0.96^(88%)^**1.80	1.80^(310%)^**7.38	3.04^(−12%)^2.69	2.69^(6%)^2.84	16.25^(−86%)^**2.29	2.29^(−68%)^**0.73
Jiangsu	3.99^(−54%)^**1.83	1.83^(−54%)^**0.84	18.40^(1%)^18.60	18.60^(−3%)^17.95	0.81^(190%)^**2.35	2.35^(69%)^**3.97	1.49^(183%)^**4.22	4.22^(−10%)^3.81	10.37^(−51%)^**5.12	5.12^(−42%)^**2.98
Zhejiang	7.40^(−71%)^**2.11	2.11^(−60%)^**0.84	66.17^(−2%)^64.83	64.83^(−64%)^**23.57	0.86^(340%)^**3.78	3.78^(28%)^4.82	2.41^(55%)^3.73	3.73^(−15%)^3.16	12.79^(−38%)^**7.96	7.96^(−68%)^**2.56
Anhui	5.75^(−70%)^**1.74	1.74^(−43%)^**0.99	44.72^(18%)^**52.58	52.58^(17%)^*61.28	0.57^(509%)^**3.47	3.47^(200%)^**10.40	0.33^(588%)^**2.27	2.27^(15%)^2.61	3.98^(−4%)^3.82	3.82^(1%)^3.87
Fujian	6.12^(−51%)^**2.99	2.99^(−53%)^**1.42	76.61^(89%)^**144.92	144.92^(−11%)^*129.41	0.38^(1192%)^**4.91	4.91^(53%)^**7.50	0.30^(510%)^**1.83	1.83^(37%)^2.50	21.21^(−29%)^**15.02	15.02^(−55%)^**6.76
Jiangxi	8.84^(−66%)^**3.03	3.03^(−80%)^**0.61	59.54^(37%)^81.33	81.33^(5%)^85.23	0.50^(528%)^**3.14	3.14^(165%)^**8.32	0.22^(386%)^**1.07	1.07^(70%)^1.82	4.96^(−18%)^4.09	4.09^(−44%)^**2.29
Shandong	2.45^(−78%)^**0.55	0.55^(−4%)^0.53	31.89^(12%)^35.65	35.65^(37%)^**48.75	0.30^(423%)^**1.57	1.57^(169%)^**4.22	0.91^(40%)^1.27	1.27^(−3%)^1.23	3.57^(−37%)^**2.26	2.26^(−44%)^**1.26
Henan	11.34^(−62%)^**4.28	4.28^(−90%)^**0.41	69.84^(141%)^**168.34	168.34^(−62%)^**63.62	2.05^(1081%)^**24.21	24.21^(8%)^26.22	0.08^(425%)^**0.42	0.42^(52%)^0.64	2.18^(−33%)^1.47	1.47^(−69%)^**0.45
Hubei	6.01^(−45%)^**3.29	3.29^(−55%)^**1.47	81.49^(71%)^**139.63	139.63^(−24%)^**106.43	1.01^(732%)^**8.40	8.40^(79%)^**15.02	0.88^(220%)^**2.82	2.82^(42%)^**4.00	5.79^(4%)^6.03	6.03^(−44%)^**3.38
Hunan	3.29^(−40%)^1.96	1.96^(−53%)^**0.92	29.67^(115%)^**63.72	63.72^(27%)^**80.70	0.74^(857%)^**7.08	7.08^(175%)^**19.45	0.10^(610%)^**0.71	0.71^(141%)^**1.71	2.23^(41%)^**3.15	3.15^(−26%)^2.33
Guangdong	2.00^(−8%)^1.85	1.85^(−16%)^1.55	29.72^(361%)^**137.10	137.10^(−2%)^134.56	0.89^(1382%)^**13.19	13.19^(49%)^**19.64	0.49^(388%)^**2.39	2.39^(7%)^2.55	3.81^(−10%)^3.43	3.43^(−37%)^**2.15
Guangxi	4.27^(−32%)^*2.92	2.92^(−48%)^**1.53	55.76^(53%)^**85.21	85.21^(2%)^87.13	2.51^(573%)^**16.89	16.89^(14%)^19.18	0.29^(338%)^**1.27	1.27^(155%)^**3.24	4.48^(−3%)^4.33	4.33^(−21%)^3.44
Hainan	5.88^(−49%)^2.99	2.99^(−70%)^**0.91	52.09^(41%)^73.35	73.35^(74%)^**127.63	0.64^(1075%)^**7.52	7.52^(393%)^**37.09	0.01^(4200%)^**0.43	0.43^(519%)^**2.66	4.55^(74%)^**7.92	7.92^(−62%)^**3.02
Chongqing	12.29^(−56%)^**5.35	5.35^(−45%)^**2.93	81.55^(−9%)^74.10	74.10^(−11%)^66.03	0.81^(616%)^**5.80	5.80^(126%)^**13.08	0.13^(354%)^**0.59	0.59^(410%)^**3.01	4.95^(−29%)^**3.49	3.49^(−64%)^**1.24
Sichuan	20.09^(−71%)^**5.84	5.84^(−50%)^**2.93	44.78^(72%)^77.10	77.10^(−39%)^**47.39	0.56^(923%)^**5.73	5.73^(113%)^**12.23	0.12^(442%)^**0.65	0.65^(123%)^*1.45	6.32^(−41%)^**3.70	3.70^(−63%)^**1.38
Guizhou	17.71^(−46%)^9.62	9.62^(−88%)^**1.11	24.57^(286%)^**94.88	94.88^(−38%)^**58.71	0.98^(556%)^**6.43	6.43^(109%)^**13.42	0.60^(−42%)^0.35	0.35^(394%)^**1.73	4.02^(−27%)^2.94	2.94^(−67%)^**0.96
Yunnan	25.98^(−67%)^*8.65	8.65^(−68%)^**2.74	13.53^(238%)^*45.75	45.75^(−1%)^45.25	0.17^(5541%)^**9.59	9.59^(129%)^**22.00	0.03^(3733%)^**1.15	1.15^(126%)^**2.60	6.17^(−81%)^**1.18	1.18^(−75%)^**0.29
Xizang	14.15^(−1%)^14.04	14.04^(−43%)^7.97	64.42^(−76%)^15.64	15.64^(403%)^**78.73	1.21^(−60%)^0.49	0.49^(100%)^0.98	0.15^(60%)^0.24	0.24^(−46%)^0.13	5.90^(−88%)^**0.73	0.73^(90%)^1.39
Shaanxi	9.60^(−73%)^**2.62	2.62^(−62%)^**1.00	127.35^(−33%)^**85.68	85.68^(−28%)^**61.38	2.82^(259%)^**10.12	10.12^(88%)^**19.05	0.18^(128%)^*0.41	0.41^(78%)^0.73	5.21^(−51%)^**2.53	2.53^(−69%)^**0.79
Gansu	18.61^(−35%)^12.15	12.15^(−76%)^**2.89	172.46^(37%)^**237.04	237.04^(−84%)^**38.78	5.95^(323%^**^)^25.17	25.17^(4%)^26.21	0.08^(263%)^**0.29	0.29^(66%)^*0.48	3.49^(−18%)^2.86	2.86^(−62%)^**1.08
Qinghai	18.64^(−37%)^11.71	11.71^(−51%)^*5.79	153.14^(170%)^**413.29	413.29^(−65%)^**146.31	3.57^(838%)^**33.47	33.47^(2%)^34.14	0.11^(536%)^**0.70	0.70^(76%)^*1.23	6.00^(−56%)^**2.63	2.63^(−49%)^*1.35
Ningxia	24.36^(−30%)^17.17	17.17^(−87%)^*2.24	130.09^(−14%)^112.19	112.19^(−61%)^**44.02	1.25^(334%)^**5.42	5.42^(124%)^**12.15	0.18^(178%)^**0.50	0.50^(−52%)^0.24	5.19^(−45%)^2.84	2.84^(−80%)^**0.56
Xinjiang	11.24^(−21%)^8.88	8.88^(86%)^16.52	50.62^(319%)^**211.91	211.91^(−22%)^*164.31	2.49^(1507%)^**40.02	40.02^(19%)^47.66	0.18^(467%)^**1.02	1.02^(123%)^**2.27	5.73^(−47%)^3.06	3.06^(−8%)^*2.81
SUM	7.37^(−55%)^**3.30	3.30^(−50%)^**1.66	53.32^(67%)^**88.82	88.82^(−23%)^**68.57	1.57^(532%)^**9.93	9.93^(54%)^**15.26	0.72^(113%)^**1.53	1.53^(30%)^1.99	5.57^(−33%)^**3.72	3.72^(−47%)^**1.98

^1^ Growth rates in parentheses, units which displayed a significant linear trend during the subperiod are in bold. * Statistical significance at 10% level; ** Statistical significance at 5% level; *** Statistical significance at 1% level.

**Table 2:** Global spatial autocorrelation analysis and test results ^2^.

Year	Hepatitis A			Hepatitis B			Hepatitis C			Hepatitis E			Unclassified Hepatitis		
	Moran’s I	Z-Value	*p*-Value	Moran’s I	Z-Value	*p*-Value	Moran’s I	Z-Value	*p*-Value	Moran’s I	Z-Value	*p*-Value	Moran’s I	Z-Value	*p*-Value
2003	0.4605	4.3622	0.0002	0.3486	3.4146	0.0029	0.5125	5.0126	0.0003	0.2093	2.3184	0.0256	0.3084	3.3259	0.0054
2004	0.5586	5.1435	0.0002	0.3801	3.6752	0.0013	0.4496	4.2313	0.0005	0.2941	3.0198	0.0083	0.3298	3.8927	0.0020
2005	0.5082	5.2675	0.0001	0.3446	3.3712	0.0017	0.3965	3.7419	0.0008	0.3620	3.5710	0.0024	0.3279	3.5401	0.0041
2006	0.5262	5.1908	0.0001	0.3172	3.1546	0.0046	0.2800	2.7817	0.0069	0.3868	3.7780	0.0011	0.3369	3.5350	0.0035
2007	0.3813	3.8694	0.0022	0.3317	3.2922	0.0027	0.2709	2.7911	0.0072	0.3577	3.3243	0.0029	0.2346	2.6270	0.0108
2008	0.5486	5.1385	0.0001	0.3186	3.2575	0.0029	0.2585	2.7350	0.0085	0.3990	3.7677	0.0009	0.2724	3.1474	0.0049
2009	0.4961	4.7851	0.0002	0.2510	2.8569	0.0066	0.2835	2.8584	0.0072	0.4314	4.1267	0.0005	0.1994	2.4228	0.0160
2010	0.4449	5.0161	0.0005	0.2881	2.9339	0.0060	0.2670	2.6532	0.0104	0.4378	4.0594	0.0003	0.1668	2.1739	0.0245
2011	0.5467	5.5616	0.0006	0.3382	3.3336	0.0028	0.2438	2.5212	0.0133	0.4265	4.1490	0.0004	0.1880	2.3237	0.0175
2012	0.6636	6.8015	0.0001	0.2644	2.6571	0.0101	0.2317	2.4156	0.0164	0.3042	2.9360	0.0044	0.1463	2.1520	0.0208
2013	0.6226	6.4308	0.0002	0.1740	1.8903	0.0420	0.2293	2.4237	0.0146	0.2898	2.8619	0.0055	0.0943	1.3990	0.0812
2014	0.2434	4.7610	0.0006	0.2247	2.2409	0.0209	0.2245	2.3661	0.0151	0.2885	2.8319	0.0064	0.1152	1.4535	0.0821
2015	0.4039	5.1859	0.0005	0.2565	2.5737	0.0097	0.2209**	2.2592	0.0199	0.3572	3.4000	0.0013	0.1402	1.5063	0.0648

^2^ Tibet was excluded in the calculation of global Moran’s I for the incidence of hepatitis E in 2004, 2008, and 2010 due to the lack of data.

**Table 3:** The mostly likely clusters and secodary clusters of viral hepatitis in China ^3^.

Type of Viral Hepatitis	Cluster Type	Location	Location IDs Included	Coordinates	Radius (Km)	Time (Year)	Number of Cases	Expected Cases	Annual Cases/100,000	RELATIVE RISK	LLR	*p*-Value
A	Most likely cluster	Xizang	Xizang, Qinghai, Xinjiang, Sichuan, Yunnan, Gansu, Ningxia, Guizhou, Chongqing	31.1 N, 89.1 E	1790.28	2003–2008	225,471	61,027.91	14.7	5.02	154,163.66	<0.001
A	Secondary cluster	Henan	Henan, Hubei, Anhui, Shaanxi	33.8 N, 113.6 E	445.66	2003–2004	40,424	20,496.61	7.8	2.03	7829.79	<0.001
B	Most likely cluster	Gansu	Gansu, Ningxia, Shaanxi, Sichuan, Qinghai, Chongqing, Shanxi, Neimenggu, Henan, Hubei	36.0 N, 103.8 E	1023.00	2006–2011	3,010,858	1,839,009.52	127.0	1.82	374,337.42	<0.001
B	Secondary cluster	Hainan	Hainan, Guangxi, Guangdong	19.2 N, 109.8 E	583.68	2010–2015	1,160,486	754,743.36	119.3	1.59	100,129.46	<0.001
B	Secondary cluster	Heilongjiang	Heilongjiang	46.8 N, 127.9 E	—	2004–2005	63,354	59,295.19	82.9	1.07	136.47	<0.001
C	Most likely cluster	Xizang	Xizang, Qinghai, Xinjiang, Sichuan, Yunnan, Gansu, Ningxia, Guizhou, Chongqing, Shaanxi, Guangxi, Hunan, Shanxi, Neimenggu, Hubei, Henan, Hainan	31.1 N, 89.1 E	2454.71	2010–2015	743,192	366,284.22	19.7	2.86	209,702.96	<0.001
E	Most likely cluster	Fujian	Fujian, Jiangxi, Zhejiang, Guangdong, Anhui, Hunan, Shanghai, Hubei, Jiangsu	26.0 N, 118.0 E	742.95	2010–2015	95,534	52769.84	3.0	2.22	18,154.26	<0.001
E	Secondary cluster	Liaoning	Liaoning	41.5 N, 123.5 E	—	2003–2008	13,013	4225.54	5.1	3.18	5988.83	<0.001
E	Secondary cluster	Chongqing	Chongqing	29.8 N, 107.8 E	—	2013–2015	2759	1483.82	3.1	1.87	438.96	<0.001
E	Secondary cluster	Xinjiang	Xinjiang	42.0 N, 85.7 E	—	2015	535	388.91	2.3	1.38	24.57	<0.001
Unspeficied	Most likely cluster	Zhejiang	Zhejiang, Shanghai, Jiangsu, Fujian	29.1 N, 120.1 E	400.88	2003–2008	129,643	43,380.75	12.1	3.44	61,614.92	<0.001
Unspeficied	Secondary cluster	Guizhou	Guizhou, Chongqing, Guangxi, Hunan, Sichuan, Yunnan, Guangdong, Hubei, Shaanxi, Hainan, Jiangxi	26. 7 N, 106.6 E	899.36	2003–2007	127,014	110,380.63	4.7	1.18	1432.19	<0.001
Unspeficied	Secondary cluster	Xinjiang	Xinjiang	42.0 N, 85.7 E	—	2004–2007	6704	3297.72	8.3	2.04	1358.39	<0.001

^3^ The radius is reported only when the cluster areas include more than 1 units, and the criteria for Reporting Secondary Clusters is “no geographical overlap”.

## Supplementary Materials

The following are available online at http://www.mdpi.com/1660-4601/15/4/661/s1, Table S1. The incidence of hepatitis A, B, C, E and unspecified in each provincial unit from 2003 to 2015. Table S2. The population in each provincial unit from 2003 to 2015. Table S3. The formulas of and detailed explanations for Global and Local Moran’s I. Table S4. The chi-square and *p*-value results for linear by linear association test.
