# Supplementary material for: Spatio-Temporal Epidemiology of Viral Hepatitis in China (2003–2015): Implications for Prevention and Control Policies
Source: Int J Environ Res Public Health. 2018 Apr 2;15(4):661. doi: 10.3390/ijerph15040661 (PMC5923703; doi:10.3390/ijerph15040661)
Supplement: Supplementary file 1 [file ijerph-15-00661-s001.zip › Supplementary materials/Table S3 The formulas of and detailed explanations for Global and Local Moran’s I.pdf]

	Global Moran's I	Local Moran's I
**Definition**	Indicator which reveals the spatial autocorrelation at the global level.	Indicator which reveals the spatial autocorrelation at the local level.
**Usage in this study**	To reveal the overall agglomerating level of the incidence of viral hepatitis across the space.	To identify the spatial clusters of the incidence of viral hepatitis across the space.
**Formula**	$\text{Global Moran's I} = \frac{n\sum_{i=1}^{n}\sum_{j=1}^{n} W_{ij}(x_i-\bar{x})(x_j-\bar{x})}{\left(\sum_{i=1}^{n}\sum_{j=1}^{n} W_{ij}\right)\sum_{i=1}^{n}(x_i-\bar{x})^2}$	$\text{Local Moran's I} = \frac{(x_i-\bar{x})}{\text{m}_0}\sum_{j} W_{ij}(x_j-\bar{x})$<br>$\text{m}_0 = \sum_{i}(x_i-\bar{x})^2/\text{n}.$
**Explanations**	x_i—the incidence of viral hepatitis in provincial unit i.<br>x_j—the incidence of viral hepatitis in provincial unit j.<br>$\bar{x}$—the mean value of the incidence of viral hepatitis at the provincial level.<br>W_{ij}—row-standardized 31×31 spatial weight matrix, which contains the geographical information of research target observation units. It is defined as follows:<br>$W_{ij} = \begin{bmatrix} W_{11} & W_{12} & \cdots & W_{1,30} & W_{1,31} \\ W_{21} & W_{22} & & W_{2,30} & W_{2,31} \\ \vdots & & W_{ij} = \begin{cases} 1 & adjacent \\ 0 & otherwise \end{cases} & & \vdots \\ W_{30,1} & W_{30,2} & \cdots & W_{30,30} & W_{30,29} \\ W_{31,1} & W_{31,2} & & W_{31,30} & W_{31,31} \end{bmatrix}$	x_i—the incidence of viral hepatitis in provincial unit i.<br>x_j—the incidence of viral hepatitis in provincial unit j.<br>j—the number of adjacent units of unit i, which indicates that the sum of j will be limited to the adjacent units of i.<br>W_{ij} shares the same meaning with Global Moran's I.
