# Supplementary material for: Spatio-Temporal Epidemiology of Viral Hepatitis in China (2003–2015): Implications for Prevention and Control Policies
Source: Int J Environ Res Public Health. 2018 Apr 2;15(4):661. doi: 10.3390/ijerph15040661 (PMC5923703; doi:10.3390/ijerph15040661)
Supplement: Supplementary file 1 [file ijerph-15-00661-s001.zip › Supplementary materials/Table S4 The chi-square and P-value results for linear by linear association test.pdf]

Table S4 The chi-square and P-value results for linear by linear association test.

Region	Hepatitis A Chi-sqaure (P-value)		Hepatitis B Chi-sqaure (P-value)		Hepatitis C Chi-sqaure (P-value)		Hepatitis E Chi-sqaure (P-value)		Hepatitis non A-E Chi-sqaure (P-value)	
	**2003-2009**	**2009-2015**	**2003-2009**	**2009-2015**	**2003-2009**	**2009-2015**	**2003-2009**	**2009-2015**	**2003-2009**	**2009-2015**
Beijing	5.676(0.017)	2.436(0.119)	0.003(0.957)	5.709(0.017)	3.826(0.050)	5.463(0.019)	0.113(0.736)	5.111(0.024)	5.348(0.021)	4.905(0.027)
Tianjin	5.172(0.023)	1.579(0.209)	0.060(0.806)	5.309(0.021)	0.896(0.344)	0.247(0.619)	3.378(0.066)	5.357(0.021)	4.573(0.032)	5.491(0.019)
Hebei	5.807(0.016)	3.396(0.065)	4.186(0.041)	0.729(0.393)	5.865(0.015)	5.935(0.015)	0.100(0.752)	1.340(0.247)	4.894(0.027)	4.983(0.026)
Shanxi	4.862(0.027)	4.363(0.037)	5.027(0.025)	0.595(0.440)	5.896(0.015)	5.373(0.020)	2.451(0.117)	4.433(0.035)	1.334(0.248)	2.946(0.086)
Neimenggu	5.220(0.022)	2.313(0.128)	4.619(0.032)	5.047(0.025)	5.980(0.014)	4.447(0.035)	0.854(0.355)	4.673(0.031)	2.029(0.154)	5.004(0.025)
Liaoning	4.880(0.027)	1.703(0.192)	4.358(0.037)	5.243(0.022)	5.908(0.015)	4.987(0.026)	3.684(0.055)	3.092(0.079)	4.781(0.029)	5.498(0.019)
Jilin	5.504(0.019)	3.941(0.047)	5.401(0.020)	5.128(0.124)	5.704(0.017)	2.432(0.119)	0.223(0.636)	0.217(0.641)	5.653(0.017)	5.198(0.023)
Heilongjiang	5.423(0.020)	4.112(0.043)	1.931(0.165)	5.693(0.017)	5.060(0.024)	0.097(0.756)	0.000(0.992)	4.639(0.031)	4.149(0.042)	5.697(0.017)
Shanghai	5.590(0.018)	2.334(0.127)	1.859(0.173)	0.950(0.330)	5.500(0.019)	4.763(0.029)	2.399(0.121)	1.057(0.304)	3.891(0.049)	5.397(0.020)
Jiangsu	4.910(0.027)	4.606(0.032)	0.804(0.370)	0.625(0.429)	4.991(0.025)	5.591(0.018)	4.435(0.035)	0.853(0.356)	5.324(0.021)	4.868(0.027)
Zhejiang	5.020(0.025)	4.778(0.029)	1.246(0.264)	5.282(0.022)	5.683(0.017)	1.175(0.278)	2.703(0.100)	2.428(0.119)	4.525(0.033)	5.489(0.019)
Anhui	5.092(0.024)	4.906(0.027)	4.267(0.039)	3.373(0.066)	5.689(0.017)	5.973(0.015)	5.416(0.020)	0.325(0.568)	0.303(0.582)	0.452(0.502)
Fujian	5.238(0.022)	4.808(0.028)	5.666(0.017)	3.218(0.073)	5.666(0.017)	5.153(0.023)	4.796(0.029)	0.389(0.533)	5.442(0.020)	4.603(0.032)
Jiangxi	5.479(0.019)	5.069(0.024)	2.299(0.129)	1.333(0.248)	5.711(0.017)	5.677(0.017)	5.476(0.019)	1.970(0.160)	2.013(0.156)	5.115(0.024)
Shandong	5.733(0.017)	0.945(0.331)	0.001(0.980)	4.812(0.028)	5.446(0.020)	5.779(0.016)	1.649(0.199)	0.029(0.864)	4.283(0.038)	5.034(0.025)
Henan	3.930(0.047)	5.274(0.022)	4.995(0.025)	5.195(0.023)	5.979(0.014)	0.099(0.753)	4.543(0.033)	2.696(0.101)	2.458(0.117)	5.672(0.017)
Hubei	5.560(0.018)	4.841(0.028)	5.700(0.017)	4.726(0.030)	5.898(0.015)	5.895(0.015)	4.714(0.030)	5.058(0.025)	0.230(0.652)	5.805(0.016)
Hunan	1.875(0.174)	4.922(0.027)	5.346(0.021)	4.982(0.026)	5.679(0.017)	5.827(0.016)	5.274(0.022)	3.968(0.046)	4.251(0.039)	2.118(0.146)
Guangdong	2.428(0.119)	0.183(0.668)	5.515(0.019)	0.026(0.872)	5.984(0.014)	4.830(0.028)	5.862(0.015)	0.318(0.573)	1.675(0.196)	3.983(0.046)
Guangxi	3.048(0.081)	4.474(0.034)	5.145(0.023)	0.019(0.891)	5.884(0.015)	0.629(0.428)	5.636(0.018)	5.490(0.019)	0.542(0.462)	2.004(0.157)
Hainan	2.542(0.111)	5.510(0.019)	0.063(0.803)	5.485(0.019)	5.802(0.016)	5.714(0.017)	5.773(0.016)	4.914(0.029)	4.261(0.039)	5.172(0.023)
Chongqing	5.699(0.017)	3.946(0.047)	0.071(0.790)	0.507(0.476)	5.711(0.017)	5.925(0.015)	4.184(0.041)	5.260(0.022)	4.559(0.033)	5.691(0.017)
Sichuan	4.783(0.029)	5.671(0.017)	0.221(0.638)	5.675(0.017)	5.648(0.017)	5.126(0.024)	4.145(0.042)	3.705(0.054)	4.348(0.037)	5.869(0.015)
Guizhou	0.946(0.331)	4.602(0.032)	5.719(0.017)	4.105(0.043)	5.496(0.019)	5.861(0.015)	0.197(0.657)	4.824(0.028)	1.138(0.286)	5.835(0.016)
Yunnan	2.823(0.093)	4.546(0.033)	3.447(0.063)	0.670(0.413)	5.950(0.015)	5.888(0.015)	4.928(0.026)	5.086(0.024)	4.212(0.040)	5.593(0.018)
Xizang	0.000(0.989)	0.135(0.713)	1.639(0.200)	5.458(0.019)	0.361(0.548)	2.622(0.105)	0.285(0.593)	0.103(0.749)	4.539(0.033)	2.327(0.127)
Shaanxi	4.873(0.027)	4.597(0.032)	3.943(0.047)	4.439(0.035)	4.712(0.030)	5.836(0.016)	3.750(0.053)	0.782(0.377)	5.075(0.024)	5.746(0.017)
Gansu	2.446(0.118)	4.612(0.032)	4.791(0.029)	5.416(0.020)	5.640(0.018)	0.114(0.736)	5.488(0.019)	2.773(0.096)	1.394(0.238)	4.247(0.039)
Qinghai	0.742(0.389)	3.810(0.051)	5.522(0.019)	4.831(0.028)	5.594(0.018)	1.843(0.175)	4.352(0.037)	2.912(0.088)	4.233(0.040)	3.664(0.056)
Ningxia	0.000(0.989)	3.653(0.056)	1.329(0.249)	4.135(0.042)	5.686(0.017)	4.774(0.029)	4.224(0.040)	1.284(0.257)	1.929(0.165)	5.048(0.025)
Xinjiang	0.018(0.894)	0.997(0.318)	5.201(0.023)	3.741(0.053)	5.847(0.016)	2.623(0.105)	4.077(0.043)	5.086(0.024)	1.948(0.163)	1.849(0.074)
SUM	5.260(0.022)	4.738(0.030)	4.916(0.027)	5.199(0.023)	5.980(0.014)	5.008(0.025)	4.025(0.045)	1.132(0.287)	4.630(0.031)	5.904(0.015)
